# NGOs, Foreign Aid, and Development in Nepal

**DOI:** 10.3389/fpubh.2016.00177

**Published:** 2016-08-24

**Authors:** Rajendra Karkee, Jude Comfort

**Affiliations:** ^1^School of Public Health and Community Medicine, BP Koirala Institute of Health Sciences, Dharan, Nepal; ^2^School of Public Health, Curtin University, Perth, WA, Australia

**Keywords:** non-governmental organization, development, aid, Nepal

## Abstract

The number of non-governmental organizations (NGOs) working in Nepal has grown significantly since the 1990s due to a range of factors. A total of 39,759 NGOs and 189 international non-governmental organizations were registered in Nepal between 1977 and 2014 in various sectors, including health, agriculture, poverty alleviation, and good governance. Despite thousands of NGOs and significant amounts of foreign aid, Nepal remains one of the poorest countries in South Asia. The case of Nepal indicates that aid and donor support alone are insufficient for sustained development.

## Background

There has been a world-wide increase in the number of non-governmental organizations (NGOs) since the 1970s, which peaked during the 1990s (1). In general, low-income countries like Nepal have weak governance; poor resources and high unemployment (2, 3). These countries have inadequate national budgets to support universal health and education coverage and rely on the support of private organizations. These countries have become fertile land for the growth of NGOs (1). In these countries, NGOs implement programs providing various services (for example, health care, education, microfinance, agricultural products, livelihood activities) or work as an advocate for empowerment, community participation, and democracy (for example gender equality, right-based approach, marginalization issues) (4). Had these governments been able to provide universal coverage in health and education, the majority of NGOs may have never established themselves. The purpose of this article is to explore the status of NGOs and foreign aid and their impact on social development in Nepal – one of the low-income countries. While Social development can be defined broadly, this article focuses on improvements in human well-being, such as income, good health, and education (5).

## NGOs in Nepal

In Nepal, NGOs started to emerge as early as 1950, and their number increased to 220 in 1990 and 1,210 in 1993 (6). The rapid increase in the number might have been favored by the restoration of a multi-party democracy in 1990 from the one-party *Panchyat* government in Nepal (7). The political ideology of a country needs to favor the functioning of NGOs, if they are to flourish (1). An NGO needs to be registered at the District Administration Office with necessary information that includes name and address, objectives of the organization, source of funding, and names of any management committee members. The organization needs to be affiliated with the Social Welfare Council of the Government of Nepal, and its programme and any foreign aid needs the approval of the Council.

According to the Social Welfare Council, there were 39,759 NGOs registered between 1977 and 2014 in Nepal (8). Table 1 gives registered NGOs during this period in different sectors. The majority of NGOs were registered in community and rural development sector, followed by youth services (8). Similarly, there were 189 international non-governmental organizations (INGOs) registered between 1977 and 2014 from 26 countries. The highest numbers were from US (53), followed by United Kingdom (29) and Germany (12). The total number of registered NGOs does not indicate that all are active and functioning because some NGOs may not continue to operate. The diversity and nature of NGOs working in low-income countries makes them difficult to categorize and define (9). However, the basic characteristics of an NGO established in any low-income country are that it should be not-for-profit and not be directly managed by and accountable to the state (10). An NGO is often self-governing and private in nature. If an NGO works in several countries, such an NGO is commonly referred as INGO.

**Table 1:** **Number of NGOs registered to Social Welfare Council between 1977 and 2014 in different sectors**.

Sectors	Number of NGOs
AIDS and abuse control	98
Child welfare	1,149
Community and rural development	25,403
Educational development	517
Environmental protection	1,451
Handicapped and disabled	758
Health services	875
Moral development	1,146
Women services	2,967
Youth services	5,395
Total	39,759

## Rise of NGOs

There are various reasons behind the rise of NGOs in a country. First, the national ideology of the ruling power in a country needs to favor NGOs functioning for them to flourish (10). If the country has a multi-party democratic system, this is more likely to support the establishment of NGOs as observed in Nepal after restoration of a multi-party democratic system in 1990 (11). Second, the neoliberal market policy that favors privatization and advocated by western countries and the World Bank have channeled increasing portions of aid and help through private organizations (12, 13). Free markets and privatization advocate that services can be provided by private sectors on a competitive basis. After the restoration of democracy, Nepal adopted a free-market policy favoring private organizations. Third is the inefficient, unrewarding, and corrupt bureaucracy that motivate alternative work mechanisms to work independently (14). Nepal’s civil system is slow to respond without a proper “punish and reward” system and with widespread corruption (14). Fourth is the alternative development paradigm that supports people-centered activities, including empowerment, rights, and participation and views government as inefficient and unable to deliver the requisite services. Instead, these services can be efficiently delivered as projects or empowerment and rights issues advocated by NGOs.

## Foreign Aid and Development in Nepal

Nepal has been receiving foreign aid for more than 60 years through foreign Government, multilaterals, and INGOs, collectively referred as external developmental partners (EDPs). Nepal is aid dependent. EDPs have been involved in Nepal’s policy making, program design, and implementation in a range of areas (15). Despite a significant injection of aid and involvement of EDPs in policy making, Nepal has failed to demonstrate significant progress during this period (14). Nepal remains one of the least-developed countries with 16.4% of the population living below US $ 1 per day (GoN 2011). Nepal’s human development index in 2011 was 0.458, the lowest among the countries of the South Asian Association for Regional Cooperation, aside from Afghanistan (16).

The important sectors receiving international aid in Nepal are education, local development, roads, and health. A brief evaluation of education and health sectors is presented below. Nepal has been receiving aid and help in the education sector since 1980 from EDPs to implement several projects: Primary Education Project (1984), Public Primary Education Project (BPEPI I, 1991–1998 and BPEP II, 1999–2003), Education for All (EFA, 2003–2009), and Community School Support Program (CSSP, 2003–2009). The latest project in the education sector that pooled resources from many external development partners was the School Sector Reform Plan (2009–2015), which received US $ 624 million over 5 years (17). EDPs have been involved in national education policy making in Nepal (18), and there was a continued loss of ownership with a lack of involvement of national stakeholders in educational reform projects (19). The policy-making process has been highly centralized with neglect at the local level and a lack of engagement of parents and communities (20).

The outputs from these educational projects were conspicuous, with many new school buildings constructed and an increase in the number of enrollments. However, the quality of education in public schools hardly changed (21). As a result, few students in public schools pass out at the 10th grade known as school leaving certificate (SLC), compared to almost all students in private schools. In 2015, 28% of students from public schools passed the SLC in Nepal, while 93% passed the same exam from private schools (22). The lower socioeconomic-status parents, especially in lower caste and marginalized communities, cannot afford private schools. Despite donor aid and support, quality education is increasingly beyond the reach of the general population in Nepal. Inequality of education was one of the reasons behind the decade-long armed conflict in Nepal, which further damaged the performance of public schools in Nepal (23).

Similarly, the health sector has received significant aid in Nepal from EDPs. About 50% of Nepal’s health budget is made up of international aid and external development partners have been involved in several health policy initiatives in Nepal. Nepal adopted the “New Nepal: Healthy Nepal” initiative in January 2009, with provision of free essential health-care packages (15). There are several challenges in the implementation of policy initiatives as well as the efficient use of aid, mainly due to a shortage of trained health workers, corruption, politicization, and inequitable access to health care (24). Further, health aid may have been poorly utilized, with resulting overlap and duplication of services and poor harmonization in Nepal (25). Malnutrition- and sanitation-related infectious diseases are leading causes of ill health (26). Out-of-pocket expenditure in health care is over 80% and is catastrophic for poor families, perpetuating poverty, and the ill-health trap (27). Universal health care is a long way off for Nepal.

A significant reduction in maternal and child mortality has been shown to result from the Safe Motherhood Program, which received significant material, technical, and policy-level involvement from EDPs (28). The Safe Motherhood Program from its inception attracted significant international funding and cooperation, notably from United States Agency for International Development (USAID) and the Department for International Development (DFID). The DFID alone provided UK £ 26 million in support to the Safe Motherhood Programme of Nepal between 1997 and 2009 (29). The funding provided for program activities, policy formulation, and infrastructure development to improve maternal health and achieve the Millenium Development Goal target. As a result, there has been a significant reduction in maternal mortality from 539/100, 000 live births to 281/100,000 live births between 1996 and 2005. However, the other important indicator of maternal health care, the skilled attendance at delivery, was just 36% in 2011, suggesting there are challenges for sustainable improvement in maternal and child health (30, 31). Further, inequity in maternal health care and maternal health gain exists among regions and ethnicities in Nepal (32). Health is largely determined by social and political factors, which have not been satisfactorily improved in Nepal, over the decades (26).

## NGOs and Government

Non-governmental organizations bring material and human resources to a country. There is expectation that these resources can help public institutions and contribute to social development and poverty alleviation. However, donor support, resources, and technical interventions alone are insufficient for sustained development (33). It is imperative that development should be initiated and sustained by the host country itself and its institutions. There is a need for good governance and a work culture in the public institutions of the host country (34). Further, a clear framework and guidelines from the government are needed to maximize the fit and output from NGOs (10). The public system in Nepal has been criticized as being poorly managed, inefficient, poorly resourced, and corrupt with an unstable government (35). In the absence of good governance and efficient institutions, there is a risk that effort of NGOs and foreign aid is being wasted and not incorporated into a country’s priority developmental plan.

## Advantages and Disadvantages of NGOs

Non-governmental organizations have been shown to be more effective as advocates when they work in a right-based approach on issues, such as empowerment, awareness, discrimination, and marginalization, than in the service provision sector (36, 37). If people are empowered and aware about their rights, they can help make their own government system work more efficiently and accountably. NGOs in Nepal have contributed significantly in areas of children’s rights, women’s rights, and girl trafficking (38). Other significant areas that NGOs can make a difference are in economic activities, including livelihood programs and agricultural support; capacity building activities, including trained human resources and infrastructure development; and environmental protection (39).

Non-governmental organizations have been criticized that their work can contribute to rather than address true sustainable development. First, NGOs came into existence mainly due to weak governance and inadequate public resources. There is the potential that they may contribute to a weaker government by imposing their own agendas and programs with little or no reference to the host country government agenda (40). If there are many NGOs, their program may overlap with each other and with that of the Government’s, making the task of developing consistent and workable policies difficult, and resulting in potential duplication of services (41). If the government is not the lead implementer, it may lose interest and ownership of the agenda. That is why, a sector-wide approach has been initiated in many recipient countries, including Nepal. In a sector-wide approach, government is the lead implementer and the resources from all organizations in a single sector are pooled into one basket. In Nepal, donors, however, are not obliged to join the pooled fund, and some have ear marked their aid for their own specific programs and associated local organizations.

Second, dependency on overseas funding may compromise NGO performance and their accountability to the people and country in which they work (1). If a NGO employs international staff, they may lack understanding of local culture and language. Third, NGOs have the potential to cause “brain drain,” as they attract efficient and talented human resources from the public system of the host country. Government employees who are more poorly paid are easily attracted to NGOs’ jobs. The difference in paid salary can be up to 20-fold higher with an NGO. This can generate inequality between government and NGO employees, resulting in demotivation on the part of government staff.

Fourth, NGOs may cease to work when their funding ends. Hence, NGO input rarely has a sustainable impact on a country but rather accomplish short-term goals. This can even make the host country and people less productive and more reliant on NGO donations, and, hence, the government may not tackle problems of their own.

## The Role of NGOs in Disasters

Relief work in the event of a disaster differs from usual developmental works. Disasters impact many people and infrastructure in a specified area. People need immediate help, and relief materials need to be quickly distributed. Governments may not have immediate resources and may not be able to reach affected communities in time. This necessitates the involvement of NGOs who can bring resources and infrastructure support to quickly distribute aid to affected communities.

In emergency relief situations of responding to a disaster, NGOs often want to work independently and donors often like to give resources directly to NGOs, bypassing the host government. This may create a difficult situation as seen in the international response to Nepal’s earthquake in April, 2015. Many NGOs wanted to work on their own, getting relief materials from the international community and choosing to go independently (42). Although distributing and working through government may not be effective and rapid in an emergency situation, there must be a co-ordination mechanism with government so that there is accountability for NGOs and there is good communication among all stakeholders to avoid duplication and to ensure the neediest are being served.

## Conclusion

There are numerous and various types of NGOs operating in diverse sectors, including health, agriculture, poverty alleviation, and good governance, in Nepal. Although NGOs and foreign aid have brought service, knowledge, and resources in Nepal producing short-term outputs, Nepal still remains one of the poorest countries in South Asia. A change in working culture with performance based evaluation in public institutions is necessary. (I)NGOs should be more involved as advocates rather than direct service providers.

## Author Contributions

RK conceived the idea of the perspective, reviewed literature, and drafted the article. JC helped in revision and interpretation of the literature. Both the authors read and approved the final version of the article.

## Conflict of Interest Statement

The authors declare that the research was conducted in the absence of any commercial or financial relationships that could be construed as a potential conflict of interest.
